# Circ_0124644 Serves as a ceRNA for miR-590-3p to Promote Hypoxia-Induced Cardiomyocytes Injury via Regulating SOX4

**DOI:** 10.3389/fgene.2021.667724

**Published:** 2021-06-25

**Authors:** Juan Tan, Weinan Pan, Huilin Chen, Yafang Du, Peiyong Jiang, Dianmei Zeng, Jie Wu, Kuang Peng

**Affiliations:** ^1^Department of Cardiology, The First Affiliated Hospital of University of South China, Hengyang, China; ^2^College of Pharmacy, Hunan Food and Drug Vocational College, Changsha, China

**Keywords:** acute myocardial infarction, hypoxia, circ_0124644, miR-590-3p, SOX4

## Abstract

Circular RNA (circRNA) is an important factor for regulating the progression of many cardiovascular diseases, including acute myocardial infarction (AMI). However, the role of circ_0124644 in AMI progression remains unclear. Hypoxia was used to induce cardiomyocytes injury. The expression of circ_0124644, microRNA (miR)-590-3p, and SRY-box transcription factor 4 (SOX4) mRNA was measured by qRT-PCR. Cell counting kit 8 (CCK8) assay and flow cytometry were utilized to detect cell viability, cell cycle progression, and apoptosis. The protein levels of apoptosis markers and SOX4 were determined by western blot (WB) analysis, and the levels of oxidative stress markers were assessed using commercial Assay Kits. Dual-luciferase reporter assay, RIP assay, and RNA pull-down assay were employed to confirm the interaction between miR-590-3p and circ_0124644 or SOX4. Circ_0124644 was upregulated in AMI patients and hypoxia-induced cardiomyocytes. Hypoxia could inhibit cardiomyocytes viability, cell cycle process, and promote apoptosis and oxidative stress, while silencing circ_0124644 could alleviate hypoxia-induced cardiomyocytes injury. In terms of mechanism, circ_0124644 could target miR-590-3p. MiR-590-3p overexpression could relieve hypoxia-induced cardiomyocytes injury. Also, the suppressive effect of circ_0124644 knockdown on hypoxia-induced cardiomyocytes injury could be reversed by miR-590-3p inhibitor. Moreover, SOX4 was found to be a target of miR-590-3p, and its overexpression also could reverse the regulation of miR-590-3p on hypoxia-induced cardiomyocytes injury. Circ_0124644 silencing could alleviate hypoxia-induced cardiomyocytes injury by regulating the miR-590-3p/SOX4 axis, suggesting that it might be a target for alleviating AMI.

## Highlights

- Circ_0124644 silencing alleviates hypoxia-induced cardiomyocytes injury.
- MiR-590-3p can be sponged by circ_0124644.
- SOX4 is a target of miR-590-3p.

## Introduction

Acute myocardial infarction (AMI) refers to myocardial necrosis caused by acute, persistent ischemia or hypoxia of coronary arteries (Simms-Thomas, 2000; Lu et al., 2015). At present, AMI is the disease with the highest fatality rate among cardiovascular diseases, and reducing its incidence and improving its prognosis are the focus of medical research (Duffy and Ferrari, 2007; Yeh et al., 2010). Many studies have confirmed that AMI induces cardiomyocyte apoptosis and oxidative stress, which in turn leads to cardiomyocyte injury (Tang et al., 2018; Huang L. et al., 2020). To clarify the molecular mechanism that affects cardiomyocyte injury and explore the molecular targets to alleviate cardiomyocyte injury is of great significance to improve the prognosis of AMI patients.

Circular RNA (circRNA) is a non-coding RNA with a special circular structure and high stability in eukaryotic cells (Li et al., 2018; Patop et al., 2019). Researchers have confirmed that circRNA can be used as a competitive endogenous RNA (ceRNA) for microRNA (miRNA) to regulate the expression of downstream targets (Ma et al., 2020). Studies have found that circRNA is closely related to the progression of a variety of cardiovascular diseases, including AMI (Altesha et al., 2019; Gong et al., 2019). Due to the high conservation and stability of circRNA, many circRNAs are gradually being discovered as potential biomarkers for cardiovascular diseases (Stêpień et al., 2018; Zhou et al., 2018). For example, circ_0010729 was found to alleviate hypoxia-induced cardiomyocytes apoptosis and might be an underlying target for AMI treatment (Lei et al., 2020). And circ_101237 silencing was discovered to protect cardiomyocytes from anoxia/reoxygenation-induced injury, which was considered as a biomarker for AMI therapy (Gan et al., 2020).

In past studies, circ_0124644 [derived from roundabout guidance receptor 2 (ROBO2) gene] had been reported to be highly expressed in the serum of coronary artery disease patients and was positively correlated with disease degree (Zhao et al., 2017). In addition, high expression of circ_0124644 had been proven to promote ox-LDL-induced human vascular endothelial cell injury (Wang G. et al., 2020). However, whether circ_0124644 participates in the development of AMI has not yet been studied. Our study used hypoxia to construct a model of cardiomyocyte injury. By clarifying the role and molecular mechanism of circ_0124644 in cardiomyocyte injury, we hope to provide exact potential targets for mitigating AMI progression.

## Materials and Methods

### Serum Samples

Thirty-one patients with AMI diagnosed by angiography were recruited from The First Affiliated Hospital of University of South China (patients with severe liver and kidney dysfunction, blood system diseases, cancer, and infectious diseases were excluded). In addition, 25 healthy control volunteers were recruited in the same hospital. The blood samples were centrifuged and the serum was collected for later use. All subjects signed a written consent. This study was approved by The First Affiliated Hospital of University of South China.

### Cell Culture and Treatments

Human cardiomyocytes (AC16) (Biovector National Typical Culture Center, Beijing, China) were cultured in DMEM medium (Gibco, Grand Island, NY, United States) containing 10% fetal bovine serum (FBS; Gibco) and 1% penicillin/streptomycin (Invitrogen, Carlsbad, CA, United States). Under normoxia condition, cells were cultured at 37°C in a normoxia incubator containing 21% O_2_ and 5% CO_2_. For hypoxia treatment, the cells were grown at 37°C in a hypoxia incubator containing 1% O_2_ and 5% CO_2_ for 24 h.

### Cell Transfection

Lentivirus short hairpin RNA (shRNA) targteing circ_0124644 (sh-circ_0124644) and its controls (sh-NC), miR-590-3p mimic and inhibitor (miR-590-3p and anti-miR-590-3p) and their controls (miR-NC and anti-NC), pcDNA SRY-box transcription factor 4 (SOX4) overexpression vector (SOX4) and its control (pcDNA) were synthesized from Ribobio (Guangzhou). All oligonucleotides (50 nM) and vectors (4.0 μg) were transfected into AC16 cells with Lipofectamine 3000 (Invitrogen).

### Quantitative Real-Time PCR (qRT-PCR)

RNA was extracted using trizol reagents (Invitrogen), and cDNA was synthesized with Reverse Transcription Kit (Qiagen, Duesseldorf, Germany). qRT-PCR was performed with SYBR Green Kit (Vazyme, Nanjing, China). Relative expression was calculated by 2^–ΔΔ^ ^CT^ method with β-actin or U6 as endogenous control. All primer sequences were shown as follows: circ_0124644, F 5′-AAACTGCCTTTTGGATTTGG-3′, R 5′-CTGGCCGGTGAGATACAAGT-3′; ROBO2, F 5′-CGAGC CCACGACTCTGAAC-3′, R 5′-ACACAAACGTAGCTTCCT TCATC-3′; miR-590-3p, F 5′-GCCGAGTAATTTTATGTATAA-3′, R 5′-ATCCAGTGCAGGGTCCGAGG-3′; SOX4, F 5′-AGCG ACAAGATCCCTTTCATTC-3′, R 5′-CGTTGCCGGACTTCAC CTT-3′; β-actin, F 5′-CTCCATCCTGGCCTCGCTGT-3′, R 5′-G CTGTCACCTTCACCGTTCC-3′; U6, F 5′-ATTGGAACGAT ACAGAGAAGATT-3′, R 5′-GGAACGCTTCACGAATTTG-3′.

### RNase R Assay

After RNA was extracted from AC16 cells, the RNA was treated with RNase R (Geneseed, Guangzhou, China) for 30 min. The expression of circ_0124644 and linear ROBO2 was then measured by qRT-PCR. Non-treated RNA was used as control (Mock).

### Actinomycin D (Act D) Assay

AC16 cells were incubated with Act D solution (Bioss, Beijing, China) for 1 h. Then, the cells were further cultured for indicated times. qRT-PCR was utilized for measuring circ_0124644 and linear ROBO2 expression.

### Subcellular Localization Analysis

The cytoplasm RNA and nuclear RNA of AC16 cells were isolated using PARIS Kit (Ambion, Austin, TX, United States). After that, qRT-PCR was used to detect circ_0124644 expression in the cytoplasm RNA and nuclear RNA of AC16 cells. U6 and GAPDH expression were used as nuclear control and cytoplasm control.

### Cell Viability Assay

After transfection and treatment, AC16 cells were collected and then re-seeded into 96 well plates. Then, 48 h later, the cells were incubated with cell counting kit 8 (CCK8) solution (Apexbio, Houston, TX, United States) for 4 h. The absorbance at 450 nm was observed under a microplate reader (Bio-Rad, Hercules, CA, United States).

### Cell Cycle and Apoptosis Analysis

AC16 cells were harvested and collected into centrifuge tubes. Cell Cycle Detection Kit (KeyGen, Nanjing, China) was used for detecting cell cycle process. Briefly, cells were treated with 70% ethanol, and then incubated with propidium iodide and RNase A. The cell cycle distribution was analyzed by flow cytometer (Beckman Coulter, Miami, FL, United States). Annexin V-FITC Apoptosis Detection Kit (Beyotime, Shanghai, China) was used to measure cell apoptosis. In brief, the cell suspensions were incubated with Annexin V-FITC and propidium iodide. Finally, cell apoptosis rate was detected by flow cytometer (Beckman Coulter).

### Western Blot (WB) Analysis

RIPA buffer (Beyotime) was utilized for extracting total protein from serum and cells. Then, the protein samples were separated by 10% SDS-PAGE gel and transferred to PVDF membranes (Millipore, Billerica, MA, United States). After that, the membranes were incubated with anti-CyclinD1 (1:2,000, bs-20596R, Bioss), anti-Cleaved-caspase3 (anti-Cleaved-casp3, 1:2,000, bs-20364R, Bioss), anti-SOX4 (1:1,000, bs-11208R, Bioss), or anti-β-actin (1:20,000, bs-0061R, Bioss) at 4°C overnight. Goat Anti-rabbit IgG antibody (1:2,000, bs-0295G, Bioss) was used to incubate with the membranes for 2 h. After that, protein signal was detected by BeyoECL Plus reagent (Beyotime).

### Detection of Oxidative Stress Markers

Lactate dehydrogenase (LDH), malondialdehyde (MDA), superoxide dismutase (SOD), and catalase (CAT) commercial Assay Kits were bought from Nanjing Jiancheng Bioengineering Institute (Nanjing, China). The culture medium of AC16 cells was collected and the supernatants were obtained after centrifuged. According to the kit instructions, the levels of LDH, MDA, SOD, and CAT were examined.

### Dual-Luciferase Reporter Assay

The wild-type (wt) and mutate-type (mut) sequences of circ_0124644 or SOX4 3′UTR were cloned into psiCHECK-2 vector (Promega, Madison, WI, United States) and synthesized by General Biosystems (Anhui, China). In addition, 293T cells (Biovector National Typical Culture Center) were transfected with circ_0124644 wt/mut vector (or SOX4 3′UTR wt/mut vector) and miR-590-3p mimic (or miR-NC). Then, relative luciferase activity was measured by Dual Luciferase Reporter Gene Assay Kit (Beyotime).

### RIP Assay

Magna RIP Kit (Millipore) was utilized for this assay. AC16 cells were lysed by RIP buffer, and then the cell lysates were incubated with magnetic beads conjugated with human Ago2 antibody or IgG antibody at 4°C overnight. After the RNAs were purified, the circ_0124644 and miR-590-3p expression was analyzed by qRT-PCR.

### RNA Pull-Down Assay

According to the binding sites of circ_0124644 on miR-590-3p, the wt and mut sequences of miR-590-3p were designed. Biotin-labeled miR-590-3p-wt/mut probes (Bio-miR-590-3p-wt/mut) and control probe (Bio-NC) were synthesized by Sangon Biotech (Shanghai, China). These probes were transfected into AC16 cells for 24 h. After that, cell lysates were collected and then streptavidin-labeled magnetic beads (Pierce, Rockford, IL, United States) were added into cell lysates overnight at 4°C. Finally, circ_0124644 enrichment was detected by qRT-PCR.

### Statistical Analysis

All data were expressed as mean ± standard deviation from 3 independent experiments. GraphPad Prism 8.0 (GraphPad, San Diego, CA, United States) was utilized for data analysis. Differences were calculated using Student’s *t*-test or analyses of variance followed by Tukey *post hoc* test. The correlations among circ_0124644, miR-590-3p, and SOX4 were adopted using Pearson correlation analysis. *P* < 0.05 was considered significant difference.

## Results

### Circ_0124644 Was Upregulated in AMI Patients and Hypoxia-Induced AC16 Cells

**FIGURE 1:**
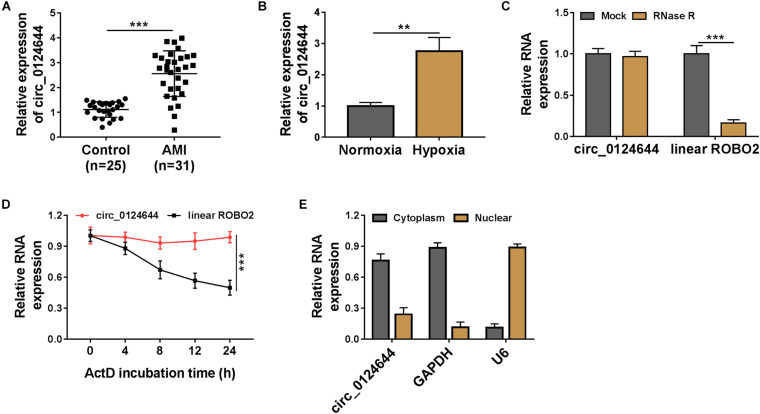
The expression of circ_0124644 in AMI patients and hypoxia-induced AC16 cells. **(A)** The circ_0124644 expression in the serum of AMI patients and healthy control volunteers was measured using qRT-PCR. **(B)** The expression of circ_0124644 was detected using qRT-PCR in hypoxia-treated and normoxia-treated AC16 cells. RNase R assay **(C)** and Act D assay **(D)** were used to confirm the stability of circ_0124644. **(E)** Subcellular localization analysis was performed to assess the distribution of circ_0124644 in the cytoplasm and nuclear of AC16 cells. **P < 0.01, ***P < 0.001.

In the serum of AMI patients, circ_0124644 was found to be highly expressed compared with that in the serum of healthy control volunteers (Figure 1A). Under the condition of hypoxia, circ_0124644 expression was significantly increased in AC16 cells (Figure 1B). RNase R assay and Act D assay were used to confirm the circular characteristic of circ_0124644 and the results suggested that circ_0124644 could resist the digestion of RNase R, and its stability was higher than linear ROBO2 mRNA (Figures 1C,D). In addition, subcellular localization analysis showed that circ_0124644 was mainly distributed in the cytoplasm of AC16 cells (Figure 1E). All data confirmed that circ_0124644 was a stable and highly expressed circRNA in AMI patients and hypoxia-induced cardiomyocytes injury models, and it might be mainly involved in post-transcriptional regulation.

### Knockdown of circ_0124644 Relieved Hypoxia-Induced Cardiomyocytes Injury

**FIGURE 2:**
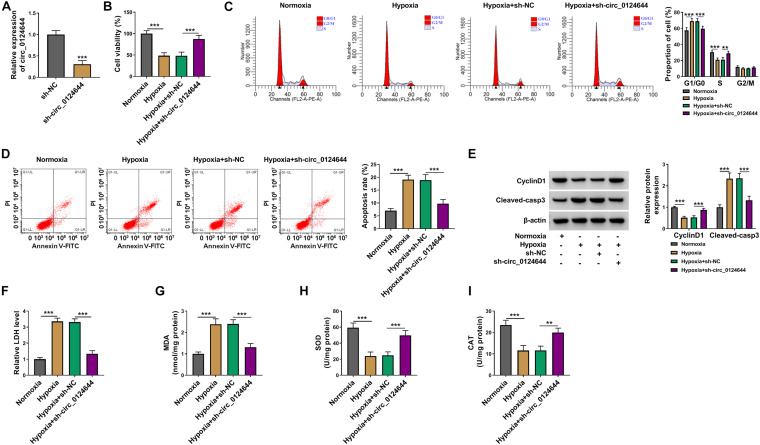
The regulation of circ_0124644 silencing on hypoxia-induced cardiomyocytes injury. **(A)** AC16 cells were transfected with sh-NC or sh-circ_0124644. The expression of circ_0124644 was measured by qRT-PCR. **(B–I)** AC16 cells were transfected with sh-NC or sh-circ_0124644, followed by treated with hypoxia. AC16 cells treated with normoxia were used as control. **(B)** CCK8 assay was used to examine cell viability. **(C,D)** Flow cytometry was performed to detect cell cycle distribution and cell apoptosis. **(E)** The protein levels of CyclinD1 and Cleaved-casp3 were determined using WB analysis. **(F–I)** Corresponding Assay Kits were used to assess the levels of LDH, MDA, SOD, and CAT. **P < 0.01, ***P < 0.001.

To explore the role of circ_0124644 in hypoxia-induced cardiomyocytes injury, the shRNA of circ_0124644 was constructed. After transfected with sh-circ_0124644 into AC16 cells, circ_0124644 expression was markedly reduced, indicating that the transfection efficiency of sh-circ_0124644 was good and could be used for further experiments (Figure 2A). Subsequently, sh-NC or sh-circ_0124644 was transfected into AC16 cells and then treated with hypoxia. By detecting cell viability, cell cycle process, and apoptosis, we discovered that hypoxia could inhibit cell viability, induce cell cycle arrest in G0/G1 phase, and enhance cell apoptosis rate. However, the cell phenotype induced by hypoxia could be reversed by circ_0124644 knockdown (Figures 2B–D). Also, hypoxia reduced the cell cycle marker CyclinD1 protein expression and promoted apoptosis marker Cleaved-casp3 protein expression in AC16 cells, while these effects could be abolished by silencing circ_0124644 (Figure 2E). Additionally, we also evaluated the levels of oxidative stress markers, and the results showed that the promotion effect of hypoxia on LDH and MDA levels and the inhibition effect on SOD and CAT levels in AC16 cells also could be overturned by circ_0124644 downregulation (Figures 2F–I). These results showed that hypoxia indeed induced cardiomyocytes injury, while silenced circ_0124644 could alleviate hypoxia-induced cardiomyocytes injury.

### Circ_0124644 Acted as a Sponge of miR-590-3p

**FIGURE 3:**
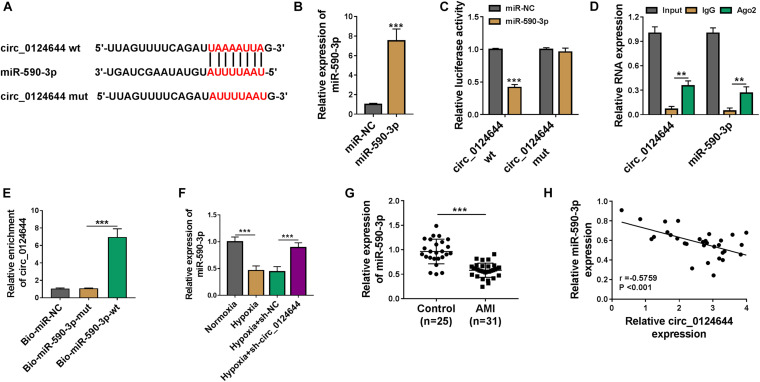
Circ_0124644 acted as a sponge of miR-590-3p. **(A)** The predicted and mutated binding sites between miR-590-3p and circ_0124644 were exhibited. **(B)** The transfection efficiency of miR-590-3p mimic was confirmed by detecting miR-590-3p expression in AC16 cells using qRT-PCR. Dual-luciferase reporter assay **(C)**, RIP assay **(D)**, and RNA pull-down assay **(E)** were employed to assess the interaction between miR-590-3p and circ_0124644. **(F)** AC16 cells were transfected with sh-NC or sh-circ_0124644, followed by treated with hypoxia. AC16 cells treated with normoxia were used as control. The expression of miR-590-3p was examined by qRT-PCR. **(G)** The expression of miR-590-3p in the serum of AMI patients and healthy control volunteers was determined by qRT-PCR. **(H)** Pearson correlation analysis was used to assess the correlation between miR-590-3p and circ_0124644. **P < 0.01, ***P < 0.001.

For investigating the new mechanism of circ_0124644 regulated cardiomyocytes injury, the circBank software^1^ was used to predict the targeted miRNA of circ_0124644. MiR-590-3p was discovered to have targeted binding sites with circ_0124644 (Figure 3A). Then, miR-590-3p mimic was established and its transfection efficiency was confirmed by detecting its high expression after transfection (Figure 3B). To further confirm the interaction between circ_0124644 and miR-590-3p, dual-luciferase reporter assay, RIP assay, and RNA pull-down assay were performed. The results suggested that the luciferase activity of the circ_0124644 wt vector rather than that of the circ_0124644 mut vector could be reduced by miR-590-3p overexpression (Figure 3C). RIP assay results manifested that both circ_0124644 and miR-590-3p were abundant in the RNA-induced silencing complex immunoprecipitated by Ago2 antibody instead of IgG antibody, implying that circ_0124644 and miR-590-3p co-existed in RNA-induced silencing complex (Figure 3D). Furthermore, the enrichment of circ_0124644 was markedly increased in the Bio-miR-590-3p-wt probe (Figure 3E). In addition, we found that hypoxia could decrease miR-590-3p expression, while this effect could be reversed by silencing circ_0124644 (Figure 3F). Compared to healthy control volunteers, miR-590-3p was remarkably downregulated in the serum of AMI patients (Figure 3G), and its expression also was negatively correlated with circ_0124644 expression in AMI patients (Figure 3H). These data confirmed that miR-590-3p could be sponged by circ_0124644.

### MiR-590-3p Overexpression Alleviated Cardiomyocytes Injury Induced by Hypoxia

**FIGURE 4:**
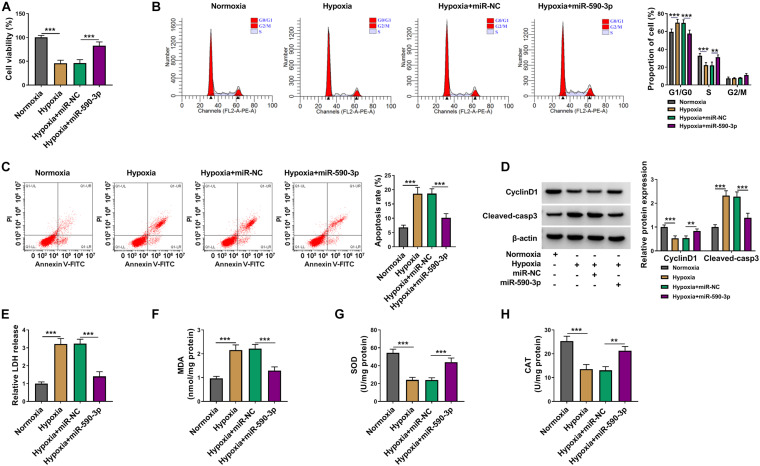
The regulation of miR-590-3p overexpression on cardiomyocytes injury induced by hypoxia. AC16 cells were transfected with miR-NC or miR-590-3p mimic, followed by treated with hypoxia. Normoxia-treated AC16 cells were used as control. **(A)** Cell viability was determined using CCK8 assay. **(B,C)** Cell cycle distribution and cell apoptosis were detected by flow cytometry. **(D)** WB analysis was performed to evaluate the protein levels of CyclinD1 and Cleaved-casp3. **(E–H)** The levels of LDH, MDA, SOD, and CAT were determined using corresponding Assay Kits. **P < 0.01, ***P < 0.001.

To confirm the function of miR-590-3p on hypoxia-induced cardiomyocytes injury, we transfected with miR-590-3p mimic into hypoxia-induced AC16 cells. Function experiments showed that overexpressed miR-590-3p could reverse the inhibitory effect of hypoxia on cell viability and cell cycle, as well as the promoting effect on cell apoptosis (Figures 4A–C). Moreover, the decreased CyclinD1 protein expression and the increased Cleaved-casp3 protein level in AC16 cells under hypoxia treatment also could be reversed by miR-590-3p overexpression (Figure 4D). Furthermore, the addition of miR-590-3p mimic also abolished the enhancing effect of hypoxia on LDH and MDA levels and the reducing effect on SOD and CAT levels in AC16 cells (Figures 4E–H). Our data showed that miR-590-3p could relieve hypoxia-induced cardiomyocytes injury.

### MiR-590-3p Inhibitor Reversed the Regulation of circ_0124644 Silencing on Hypoxia-Induced Cardiomyocytes Injury

**FIGURE 5:**
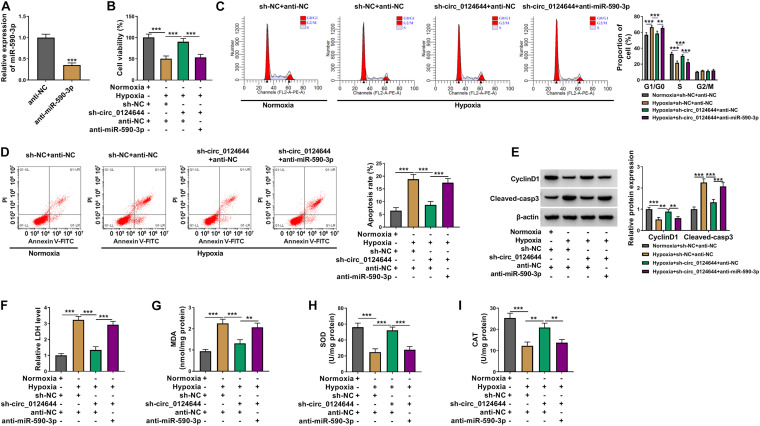
Effects of miR-590-3p inhibitor and circ_0124644 silencing on hypoxia-induced cardiomyocytes injury. **(A)** AC16 cells were transfected with anti-NC or anti-miR-590-3p. MiR-590-3p expression was detected by qRT-PCR. **(B–I)** AC16 cells were transfected with sh-NC + anti-NC, sh-circ_0124644 + anti-NC, sh-circ_0124644 + anti-miR-590-3p, followed by treated with hypoxia. Normoxia-treated AC16 cells transfected with sh-NC + anti-NC were used as control. **(B)** CCK8 assay was employed to measure cell viability. **(C,D)** Cell cycle distribution and cell apoptosis were evaluated using flow cytometry. **(E)** The protein levels of CyclinD1 and Cleaved-casp3 were examined by WB analysis. **(F–I)** The levels of LDH, MDA, SOD, and CAT were analyzed by corresponding Assay Kits. **P < 0.01, ***P < 0.001.

In order to determine that circ_0124644 regulated cardiomyocytes injury by sponging miR-590-3p, the miR-590-3p inhibitor was constructed. After transfection with anti-miR-590-3p into AC16 cells, the expression of miR-590-3p was markedly reduced, indicating that the transfection of anti-miR-590-3p was successful (Figure 5A). In hypoxia-induced AC16 cells transfected with sh-circ_0124644 and anti-miR-590-3p, we found that the promotion effect of circ_0124644 silencing on cell viability and cell cycle as well as the suppressive effect on cell apoptosis rate in hypoxia-induced AC16 cells could be reversed by miR-590-3p inhibitor (Figures 5B–D). Also, miR-590-3p inhibitor could abolish the positive regulation of circ_0124644 knockdown on CyclinD1 protein expression and the negative regulation on Cleaved-casp3 protein expression in hypoxia-induced AC16 cells (Figure 5E). In addition, the inhibiting effect of circ_0124644 silencing on LDH and MDA levels and the enhancing effect on SOD and CAT levels in hypoxia-induced AC16 cells also could be reversed by miR-590-3p inhibitor (Figures 5F–I). Therefore, we confirmed that circ_0124644 sponged miR-590-3p to regulate hypoxia-induced cardiomyocytes injury.

### SOX4 Could Be Targeted by miR-590-3p

**FIGURE 6:**
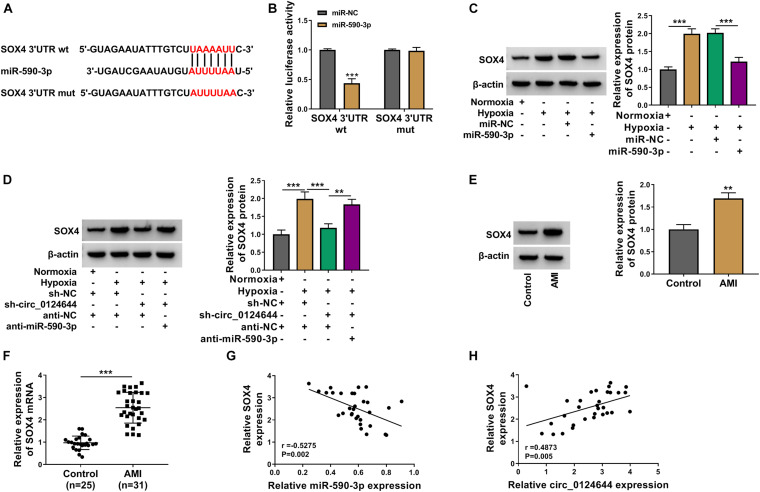
SOX4 could be targeted by miR-590-3p. **(A)** The predicted and mutated binding sites between SOX4 3′UTR and miR-590-3p were shown. **(B)** Dual-luciferase reporter assay was used to confirm the interaction between SOX4 and miR-590-3p. **(C)** AC16 cells were transfected with miR-NC or miR-590-3p mimic, followed by treated with hypoxia. AC16 cells treated with normoxia were used as control. The protein expression of SOX4 was analyzed using WB analysis. **(D)** AC16 cells were transfected with sh-NC + anti-NC, sh-circ_0124644 + anti-NC, sh-circ_0124644 + anti-miR-590-3p, followed by treated with hypoxia. Normoxia-treated AC16 cells transfected with sh-NC + anti-NC were used as control. WB analysis was used to detect SOX4 protein expression. **(E,F)** The protein and mRNA expression levels of SOX4 in the serum of AMI patients and healthy control volunteers were evaluated using WB analysis and qRT-PCR. **(G,H)** The correlation between SOX4 and miR-590-3p or circ_0124644 was assessed by Pearson correlation analysis. **P < 0.01, ***P < 0.001.

The downstream target of miR-590-3p was predicted using targetscan software^2^. It was found that the 3′UTR of SOX4 could interact with miR-590-3p (Figure 6A). Then, the results of dual-luciferase reporter assay showed that miR-590-3p mimic could inhibit the luciferase activity of SOX4 3′UTR wt vector, which had no effect on that of the SOX4 3′UTR mut vector (Figure 6B). Moreover, hypoxia could promote SOX4 expression, while this effect could be abolished by miR-590-3p overexpression (Figure 6C). Also, circ_0124644 silencing could reduce SOX4 protein expression in hypoxia-induced AC16 cells, and the addition of anti-miR-590-3p also could reverse this effect (Figure 6D). In addition, SOX4 protein and mRNA expression levels were found to be markedly enhanced in the serum of AMI patients (Figures 6E,F). Correlation analysis showed that SOX4 mRNA expression also was negatively correlated with miR-590-3p expression and positively correlated with circ_0124644 expression in AMI patients (Figures 6G,H). All data suggested that circ_0124644 sponged miR-590-3p to regulate SOX4.

### SOX4 Overexpression Reversed the Regulation of miR-590-3p on Hypoxia-Induced Cardiomyocytes Injury

**FIGURE 7:**
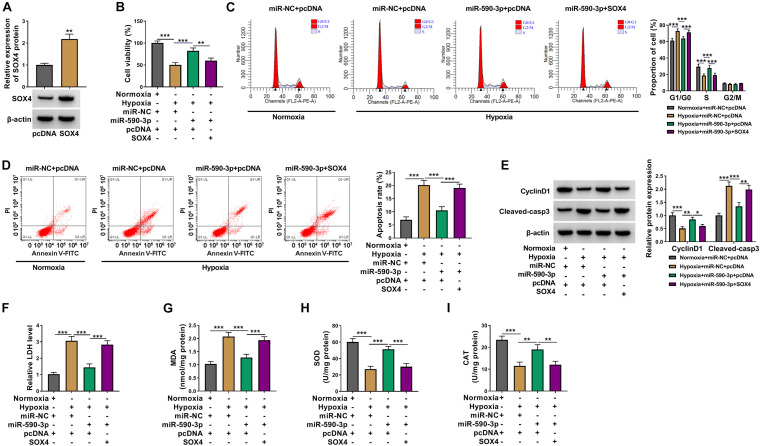
Effects of SOX4 and miR-590-3p overexpression on hypoxia-induced cardiomyocytes injury. **(A)** WB analysis was used to detect SOX4 protein expression in AC16 cells to evaluate the transfection efficiency of pcDNA SOX4 overexpression vector. **(B–I)** AC16 cells were transfected with miR-NC + pcDNA, miR-590-3p + pcDNA, or miR-590-3p + SOX4, followed by treated with hypoxia. Normoxia-treated AC16 cells transfected with miR-NC + pcDNA were used as control. **(B)** Cell viability was detected by CCK8 assay. **(C,D)** Flow cytometry was utilized for assessing cell cycle distribution and cell apoptosis. **(E)** WB analysis was used to test the protein levels of CyclinD1 and Cleaved-casp3. **(F–I)** Corresponding Assay Kits were employed to detect the levels of LDH, MDA, SOD, and CAT. *P < 0.05, **P < 0.01, ***P < 0.001.

Subsequently, pcDNA SOX4 overexpression vector was constructed. After transfection with pcDNA SOX4 overexpression vector into AC16 cells, SOX4 protein expression was significantly increased (Figure 7A). Then, miR-590-3p mimic and pcDNA SOX4 overexpression vector were co-transfected into AC16 cells, and then the cells were treated with hypoxia. Analysis results showed that ectopic SOX4 expression could reverse the enhancing effect of miR-590-3p overexpression on cell viability and cell cycle and the decreasing effect on cell apoptosis in hypoxia-induced AC16 cells (Figures 7B–D). Meanwhile, the promotion of miR-590-3p on CyclinD1 protein expression and the repression on Cleaved-casp3 protein expression also could be reversed by SOX4 upregulation in hypoxia-induced AC16 cells (Figure 7E). At the same time, overexpressed SOX4 also could abolish the miR-590-3p inhibiting LDH and MDA levels and miR-590-3p accelerating SOD and CAT levels in hypoxia-induced AC16 cells (Figures 7F–I). Hence, our results illuminated that miR-590-3p targeted SOX4 to regulate hypoxia-induced cardiomyocytes injury.

## Discussion

Cardiomyocytes apoptosis and oxidative stress are common phenomena caused by a variety of cardiovascular diseases including AMI. Therefore, inhibition of cardiomyocytes apoptosis and oxidative stress is an important measure for the targeted therapy of AMI. In the previous study, circ_0124644 was proven to be a diagnostic marker for coronary artery disease (Zhao et al., 2017) and was confirmed to promote atherosclerotic progression (Wang G. et al., 2020). In this study, a significant high circ_0124644 expression was found in the serum of AMI patients and hypoxia-induced cardiomyocytes. Further assay confirmed the stability of circ_0124644, which suggested that circ_0124644 indeed had a circular structure. In addition, the characteristic of being mainly distributed in the cytoplasm provided sufficient and necessary conditions for circ_0124644 to become the ceRNA of miRNA. By detecting cardiomyocytes viability, cell cycle process, apoptosis, and oxidative stress, we confirmed that the AMI cell model of cardiomyocytes injury *in vitro* was successful. Function experiments showed that knockdown of circ_0124644 could alleviate cardiomyocytes injury induced by hypoxia, showing that circ_0124644 downregulation might be an effective way to treat AMI.

Further analysis revealed that circ_0124644 could act as miR-590-3p ceRNA. In the past reports, miR-590-3p was confirmed to participate in cancer progression as a tumor suppressor, including hepatocellular carcinoma (Pu et al., 2020), breast cancer (Shan et al., 2020), and papillary thyroid carcinoma (Tong et al., 2019). Wang et al. showed that miR-590-3p silencing inhibited cardiomyocytes proliferation and differentiation, while it accelerated apoptosis (Wang F. et al., 2020). More importantly, miR-590-3p was found to restore cell cycle process to promote cardiomyocytes proliferation, and thus alleviating AMI progression (Wang et al., 2018). Also, Huang et al. reported that miR-590-3p was underexpressed in ischemia/reperfusion-induced cardiomyocytes and had a protective effect on cardiomyocytes (Huang P. et al., 2020). Consistent with these reports (Wang et al., 2018; Huang P. et al., 2020), our results confirmed that miR-590-3p mimic could relieve hypoxia-induced cardiomyocytes injury. The rescue experiments confirmed that the inhibitor of miR-590-3p reversed the inhibition effect of circ_0124644 knockdown on hypoxia-induced cardiomyocytes injury, revealing that circ_0124644 act as a ceRNA for miR-590-3p to regulate cardiomyocytes injury.

SOX4 belongs to the SOX transcription factor family, which plays an essential role in nervous system development, bone formation, and tumorigenesis (Cheung et al., 2000; Takahata et al., 2019; Hanieh et al., 2020). More and more studies have shown that SOX4 can participate in the development of malignant cancers such as breast cancer and gastric cancer by affecting cell growth, metastasis, and apoptosis (Ding et al., 2019; Song et al., 2019). A recent study showed that SOX4 expression was elevated in H_2_O_2_-induced cardiomyocytes, and its downregulation had a protective effect on cardiomyocytes injury (Zhang et al., 2021). Here, SOX4 was confirmed to be targeted by miR-590-3p. In addition, we discovered that SOX4 had increased expression in AMI patients and hypoxia-induced cardiomyocytes, which was consistent with the expression trend of circ_0124644 and contrary to the expression trend of miR-590-3p. The reversal effect of SOX4 on miR-590-3p regulating hypoxia-induced cardiomyocytes revealed that miR-590-3p alleviated cardiomyocytes injury via targeting SOX4. These data perfected the hypothesis of circ_0124644/miR-590-3p/SOX4 axis and proposed a new network to regulate AMI progression.

In general, our study showed that knockdown of circ_0124644 had a protective effect on hypoxia-induced cardiomyocytes injury, which was mainly achieved by mediating the miR-590-3p/SOX4 axis. Our results suggested that circ_0124644 might be a potential target for AMI treatment.

## Data Availability Statement

The datasets used and/or analyzed during the current study are available from the corresponding author on reasonable request.

## Ethics Statement

The studies involving human participants were reviewed and approved by the First Affiliated Hospital of University of South China. The patients/participants provided their written informed consent to participate in this study.

## Author Contributions

JT and KP designed the study. WP and HC analyzed the data. YD and PJ performed the experiments. DZ and JW summarized the data and wrote the manuscript. All authors contributed to this study, read, and approved the manuscript.

## Conflict of Interest

The authors declare that the research was conducted in the absence of any commercial or financial relationships that could be construed as a potential conflict of interest.

## Footnotes

1 http://www.circbank.cn/

2 http://www.targetscan.org/vert_72/

## References

[B1] Altesha M. A. Ni T. Khan A. Liu K. Zheng X. (2019). Circular RNA in cardiovascular disease. *J. Cell. Physiol.* 234 5588–5600. 10.1002/jcp.27384 30341894

[B2] Cheung M. Abu-Elmagd M. Clevers H. Scotting P. J. (2000). Roles of Sox4 in central nervous system development. *Brain Res. Mol. Brain Res.* 79 180–191. 10.1016/s0169-328x(00)00109-1 10925158

[B3] Ding L. Zhao Y. Dang S. Wang Y. Li X. Yu X. (2019). Circular RNA circ-DONSON facilitates gastric cancer growth and invasion via NURF complex dependent activation of transcription factor SOX4. *Mol. Cancer* 18:45. 10.1186/s12943-019-1006-2 30922402 PMC6437893

[B4] Duffy K. J. Ferrari V. A. (2007). Prognosis following acute myocardial infarction: insights from cardiovascular magnetic resonance. *Curr. Cardiol. Rep.* 9 57–62. 10.1007/s11886-007-0011-z 17362686

[B5] Gan J. Yuan J. Liu Y. Lu Z. Xue Y. Shi L. (2020). Circular RNA_101237 mediates anoxia/reoxygenation injury by targeting let7a5p/IGF2BP3 in cardiomyocytes. *Int. J. Mol. Med.* 45 451–460. 10.3892/ijmm.2019.4441 31894303 PMC6984805

[B6] Gong X. Wu G. Zeng C. (2019). Role of circular RNAs in cardiovascular diseases. *Exp. Biol. Med.* 244 73–82. 10.1177/1535370218822988 30654635 PMC6405821

[B7] Hanieh H. Ahmed E. A. Vishnubalaji R. Alajez N. M. (2020). SOX4: epigenetic regulation and role in tumorigenesis. *Semin. Cancer Biol.* 67 91–104. 10.1016/j.semcancer.2019.06.022 31271889

[B8] Huang L. Guo B. Liu S. Miao C. Li Y. (2020). Inhibition of the LncRNA Gpr19 attenuates ischemia-reperfusion injury after acute myocardial infarction by inhibiting apoptosis and oxidative stress via the miR-324-5p/Mtfr1 axis. *IUBMB Life* 72 373–383. 10.1002/iub.2187 31622017

[B9] Huang P. Yang D. Yu L. Shi Y. (2020). Downregulation of lncRNA ZFAS1 protects H9c2 cardiomyocytes from ischemia/reperfusioninduced apoptosis via the miR5903p/NFkappaB signaling pathway. *Mol. Med. Rep.* 22 2300–2306. 10.3892/mmr.2020.11340 32705215 PMC7411414

[B10] Lei D. Wang Y. Zhang L. Wang Z. (2020). Circ_0010729 regulates hypoxia-induced cardiomyocyte injuries by activating TRAF5 via sponging miR-27a-3p. *Life Sci.* 262:118511. 10.1016/j.lfs.2020.118511 33010282

[B11] Li X. Yang L. Chen L. L. (2018). The biogenesis, functions, and challenges of circular RNAs. *Mol. Cell* 71 428–442. 10.1016/j.molcel.2018.06.034 30057200

[B12] Lu L. Liu M. Sun R. Zheng Y. Zhang P. (2015). Myocardial infarction: symptoms and treatments. *Cell Biochem. Biophys.* 72 865–867. 10.1007/s12013-015-0553-4 25638347

[B13] Ma X. Liu C. Gao C. Li J. Zhuang J. Liu L. (2020). circRNA-associated ceRNA network construction reveals the circRNAs involved in the progression and prognosis of breast cancer. *J. Cell. Physiol.* 235 3973–3983. 10.1002/jcp.29291 31617204 PMC13482726

[B14] Patop I. L. Wust S. Kadener S. (2019). Past, present, and future of circRNAs. *EMBO J.* 38:e100836. 10.15252/embj.2018100836 31343080 PMC6694216

[B15] Pu J. Tan C. Shao Z. Wu X. Zhang Y. Xu Z. (2020). Long noncoding RNA PART1 promotes hepatocellular carcinoma progression via targeting miR-590-3p/HMGB2 Axis. *Onco Targets Ther.* 13 9203–9211. 10.2147/OTT.S259962 32982307 PMC7502387

[B16] Shan Q. Qu F. Yang W. Chen N. (2020). Effect of LINC00657 on apoptosis of breast cancer cells by regulating miR-590-3p. *Cancer Manag. Res.* 12 4561–4571. 10.2147/CMAR.S249576 32606949 PMC7305342

[B17] Simms-Thomas F. (2000). Myocardial infarction. *Clin. J. Oncol. Nurs.* 4 141–142, 144. 11235254

[B18] Song Z. Zhang X. Lin Y. Wei Y. Liang S. Dong C. (2019). LINC01133 inhibits breast cancer invasion and metastasis by negatively regulating SOX4 expression through EZH2. *J. Cell. Mol. Med.* 23 7554–7565. 10.1111/jcmm.14625 31557401 PMC6815803

[B19] Stêpień E. Costa M. C. Kurc S. Drozdz A. Cortez-Dias N. Enguita F. J. (2018). The circulating non-coding RNA landscape for biomarker research: lessons and prospects from cardiovascular diseases. *Acta Pharmacol. Sin.* 39 1085–1099. 10.1038/aps.2018.35 29877319 PMC6289369

[B20] Takahata Y. Nakamura E. Hata K. Wakabayashi M. Murakami T. Wakamori K. (2019). Sox4 is involved in osteoarthritic cartilage deterioration through induction of ADAMTS4 and ADAMTS5. *FASEB J.* 33 619–630. 10.1096/fj.201800259R 30016600

[B21] Tang Q. Li M. Y. Su Y. F. Fu J. Zou Z. Y. Wang Y. (2018). Absence of miR-223-3p ameliorates hypoxia-induced injury through repressing cardiomyocyte apoptosis and oxidative stress by targeting KLF15. *Eur. J. Pharmacol.* 841 67–74. 10.1016/j.ejphar.2018.10.014 30336138

[B22] Tong H. Zhuang X. Cai J. Ding Y. Si Y. Zhang H. (2019). Long noncoding RNA ZFAS1 promotes progression of papillary thyroid carcinoma by sponging miR-590-3p and upregulating HMGA2 expression. *Onco Targets Ther.* 12 7501–7512. 10.2147/OTT.S209138 31571903 PMC6750857

[B23] Wang F. Zhang H. Wang C. (2020). MiR-590-3p regulates cardiomyocyte P19CL6 proliferation, apoptosis and differentiation in vitro by targeting PTPN1 via JNK/STAT/NF-kB pathway. *Int. J. Exp. Pathol.* 101 196–202. 10.1111/iep.12377 33058302 PMC7691214

[B24] Wang G. Li Y. Liu Z. Ma X. Li M. Lu Q. (2020). Circular RNA circ_0124644 exacerbates the ox-LDL-induced endothelial injury in human vascular endothelial cells through regulating PAPP-A by acting as a sponge of miR-149-5p. *Mol. Cell. Biochem.* 471 51–61. 10.1007/s11010-020-03764-0 32500475

[B25] Wang Y. Ding N. Guan G. Liu G. Huo D. Li Y. (2018). Rapid delivery of Hsa-miR-590-3p using targeted exosomes to treat acute myocardial infarction through regulation of the cell cycle. *J. Biomed. Nanotechnol.* 14 968–977. 10.1166/jbn.2018.2493 29883566

[B26] Yeh R. W. Sidney S. Chandra M. Sorel M. Selby J. V. Go A. S. (2010). Population trends in the incidence and outcomes of acute myocardial infarction. *N. Engl. J. Med.* 362 2155–2165. 10.1056/NEJMoa0908610 20558366

[B27] Zhang L. Lv L. Zheng N. Li R. Yang R. Li T. (2021). Suppression of Sox4 protects against myocardial ischemic injury by reduction of cardiac apoptosis in mice. *J. Cell. Physiol.* 236 1094–1104. 10.1002/jcp.29918 32657438

[B28] Zhao Z. Li X. Gao C. Jian D. Hao P. Rao L. (2017). Peripheral blood circular RNA hsa_circ_0124644 can be used as a diagnostic biomarker of coronary artery disease. *Sci. Rep.* 7:39918. 10.1038/srep39918 28045102 PMC5206672

[B29] Zhou Q. Zhang Z. Bei Y. Li G. Wang T. (2018). Circular RNAs as novel biomarkers for cardiovascular diseases. *Adv. Exp. Med. Biol.* 1087 159–170. 10.1007/978-981-13-1426-1_13 30259365

